# An FPGA Implementation to Detect Selective Cationic Antibacterial Peptides

**DOI:** 10.1371/journal.pone.0021399

**Published:** 2011-06-28

**Authors:** Carlos Polanco González, Marco Aurelio Nuño Maganda, Miguel Arias-Estrada, Gabriel del Rio

**Affiliations:** 1 Department of Biochemistry and Structural Biology, Instituto de Fisiología Celular, Universidad Nacional Autónoma de México, México D. F., México; 2 Universidad Politécnica de Victoria, Cd. Victoria, Tamaulipas, México; 3 Computer Science Department, Instituto Nacional de Astrofísica, Óptica y Electrónica, Puebla, Puebla, México; University of Glasgow, United Kingdom

## Abstract

Exhaustive prediction of physicochemical properties of peptide sequences is used in different areas of biological research. One example is the identification of selective cationic antibacterial peptides (SCAPs), which may be used in the treatment of different diseases. Due to the discrete nature of peptide sequences, the physicochemical properties calculation is considered a high-performance computing problem. A competitive solution for this class of problems is to embed algorithms into dedicated hardware. In the present work we present the adaptation, design and implementation of an algorithm for SCAPs prediction into a Field Programmable Gate Array (FPGA) platform. Four physicochemical properties codes useful in the identification of peptide sequences with potential selective antibacterial activity were implemented into an FPGA board. The speed-up gained in a single-copy implementation was up to 108 times compared with a single Intel processor cycle for cycle. The inherent scalability of our design allows for replication of this code into multiple FPGA cards and consequently improvements in speed are possible. Our results show the first embedded SCAPs prediction solution described and constitutes the grounds to efficiently perform the exhaustive analysis of the sequence-physicochemical properties relationship of peptides.

## Introduction

Exhaustive prediction of physicochemical properties of peptides has different applications in biology, including mass-spectrometry data analysis [1], identification of disordered regions in proteins [2], trans-membrane protein analysis [3], antibacterial peptide identification [4], among others [5]. Due to the large size of the peptide sequence space (*e.*g., for peptide sequences with 25 residues long, the number of different peptide sequences is 3.3×10^32^), the exhaustive calculation of these properties demands high-performance computing. Different technical solutions exist to address such large number of computations, that may be divided into three classes: a) local solutions (*e.g.*, concurrency, design of hardware), b) distributed solutions (*e.*g., cluster of computers, cloud-computing) and c) a combination of the previous. In any case, the most efficient technical solution to satisfy high-performance computing problems starts with an efficient local solution; such is the case of application-specific integrated circuits (ASIC). However, the number and nature of physicochemical properties may vary for different applications, making the development of ASIC too expensive. Alternatively, here we present a hardware solution using a Field Programmable Gate Array (FPGA) implementation, as a cost-effective solution for this high-performance problem. The architecture of FPGA boards can be reconfigured at the level of a hardware description language, providing further advantage in the calculation of physicochemical properties of peptides.

Antibacterial peptides constitute one of the natural defences against infectious diseases and constitute a promising new area for the discovery of new pharmaceuticals [6]. Among these antibacterial peptides there are those characterized by physicochemical properties such as being positively charged, amphiphilic and small in size [7], [8]; we refer to these peptides as cationic antibacterial peptides or CAPs. Among CAPs, there is a special class of peptides that display a selective action against bacteria and do not have any toxicity against human cells [9]; these peptides are referred here to as selective CAPs or simply SCAPs. Because of the selectivity, SCAPs have been useful in the development of novel compounds useful in the treatment of cancer [10] and is foreseeable that many other diseases could be targeted through the use of SCAPs [11], [12].

Although different experimental approaches have been described for the identification of AP [13], only one has been described for the identification of SCAPs [14]. Considering the large number of possible peptide sequences, the use of computational approaches to reduce the number of peptides to be biologically essayed is important. In this scenario, we have shown that a narrow range of values for isoelectric point, helical hydrophobic moment and AGADIR score characterize SCAPs [9]. More recently, we observed that within that range of values, only some specific combination of values render SCAP activity that has been preserved during the evolution of these peptides (our unpublished results). Considering the discrete nature of the values derived from the computation of these physicochemical properties of peptides, a thorough pharmacophore virtual screening requires an efficient computational solution. An alternative solution would be to sample the peptide sequence space; for that goal we have previously described a Hidden Markov model to identify potential SCAPs [15]. However, such approach cannot guarantee to identify pharmachophores of a given length because not every peptide sequence nor every combination of physicochemical properties values is tested in this type of approach.

In the present work we aim to develop an FPGA solution for the exhaustive prediction of SCAPs in the virtual universe of peptide sequences. We describe an adaptation of the code to predict SCAPs, design and the implementation of the code into an FPGA card. The performance of our implementation is reported and compared with a software solution.

## Methods

### Design and Implementation

All the implementations, but the one for the AGADIR property, were tested in a reconfigurable platform, based on a Xilinx Virtex II Pro FPGA; the target platform was the ADM-XPL, which is a commercial coprocessor board (Alphadata). The selected hardware platform have a configurable clock generator which allows to establish the work frequency from 20 MHz up to 100 MHz, using multiples of 10 MHz. Algorithms were modelled for parallelization and optimization in the Handel-C Hardware Description Language from Celoxica using the Design Kit Suite 5.0 – DK5 (Agility). Other tools used were: Visual C++ 6.0 (for interfacing the target platform with the host PC) and Xilinx ISE 9.2i (for generating the configuration file for the target FPGA).

Prediction of SCAPs is accomplished by considering three features of peptide sequences: propensity to be unstructured (natively unstructured), charge and amphipathicity. These three features are predicted calculating four physicochemical properties:

#### a) Mean hydrophobicity (MH)

This is the normalized mean value of the hydrophobicity over all the amino acids in a given peptide. A peptide was considered SCAP if its MH value was within the range of 0.35 to 0.55.

#### b) Mean net charge (MC)

This is determined by Equation (1), which is based on a previous report [16]:
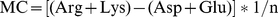
(1)The variables Arg, Lys, Asp and Glu represent the number of times the amino acids Arginine (Arg), Lysine (Lys), Aspartic acid (Asp) and Glutamic acid (Glu) appeared in the peptide sequences. The value of n is the length of the peptide.

In the present work, a peptide sequence was considered natively unfolded if the MC was above or equal to the value of C(MH):
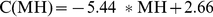
(2)


#### c) Isoelectric point (pI)

This is the pH value where a particular peptide carries no net electrical charge. A peptide was considered SCAP if it presented a pI value within the range of 10.8 to 11.8. The pI was calculated based on the following pseudo-code:

- first we count charged amino acids in a peptide sequence:
   for ( i = 0; i< = protein.length()−1; ++i)
    {
     if (protein(i) = Asp) ++AspNumber;
     if (protein(i) = Glu) ++GluNumber;
     if (protein(i) = Cys) ++CysNumber;
     if (protein(i) = Tyr) ++TyrNumber;
     if (protein(i) = His) ++HisNumber;
     if (protein(i) = Lys) ++LysNumber;
     if (protein(i) = Arg) ++ArgNumber;
    }
- Then, calculate the charge contribution from each amino acid based on the corresponding ionization constant (pKa):
   QN1 = −1/(1+10^(3.65-pH)^); //C-terminal charge
   QN2 = −AspNumber/(1+10^(3.9-pH)^); //D charge
   QN3 = −GluNumber/(1+10^(4.07-pH)^); //E charge
   QN4 = −CysNumber/(1+10^(8.18-pH)^); //C charge
   QN5 = −TyrNumber/(1+10^(10.46-pH)^); //Y charge
   QP1 = HisNumber/(1+10^(pH-6.04)^); //H charge
   QP2 = 1/(1+10^(pH-8.2)^); //NH2charge
   QP3 = LysNumber/(1+10^(pH-10.54)^); //K charge
   QP4 = ArgNumber/(1+10^(pH-12.48)^); //R charge
  NQ = QN1+QN2+QN3+QN4+QN5+QP1+QP2+QP3+QP4;
- isoelectric point is found when NQ is equal to zero. We start from pH = 0, if the result is bigger than 0, we increase pH for example of 0.01 (Assumed precision). We are doing this until NQ< = 0.

#### d) Helical hydrophobic moment (μH)

This is the sum of the hydrophobicities of the side chains of a helix of n amino acids. The length of the vector representing the hydrophobicity values is the signed numerical hydrophobicity associated with the type of side chain, and its direction is determined by the orientation of the side chain along the helix axis. A large value of μH means that the helix is perpendicular to its axis (*i.e.*, amphiphilic). A peptide was considered SCAP if at least presented an μH value within the range of 0.4 to 0.6. The helical hydrophobic moment was calculated as described previously [9] based on formula described by Eisenberg and collaborators for the hydrophobic moment plot, as described in the following pseudo-code:

- First we set the hydrophobicity for each possible amino acid.
   OMH(1) = −0.40; OMH(2) = −1.12; OMH(3) = 0.17; OMH(4) = −1.31; OMH(5) = −1.22; OMH(6) = 1.92; OMH(7) = −0.67; OMH(8) = −0.64; OMH(9) = 1.25; OMH(10) = −0.67; OMH(11) = 1.22; OMH(12) = 1.02; OMH(13) = −0.92; OMH(14) = −0.49; OMH(15) = −0.91; OMH(16) = −0.59; OMH(17) = −0.55; OMH(18) = −0.28; OMH(19) = 0.91; OMH(20) = 0.50; OMH(21) =  = 0.00; OMH(22) = 1.67; OMH(23) = −1.07; OMH(24) = 0.00;
- Calculate the necessary Angle per residue increments, (FORTRAN calculates trig functions in radians so we convert degrees to rads).
   Angle = StartAngle
   NumAngle = 0
   for ((Angle< = StopAngle) and (NumAngle< = MAXINC))
   {
    NumAngle = NumAngle+1;
    RadAngle(NumAngle) = Angle/360*2*3.14159265;
    Angle = Angle+IncAngle;
    SinSum(NumAngle) = 0;
    CosSum(NumAngle) = 0;
    CorSSum(NumAngle) = 0;
    CorCSum(NumAngle) = 0;
   }
- Calculate the hydrophobic moment and save the maximum value and corresponding angle.
- Calculate the first window.
   Hydsum = 0;
   for (Pos = Begin,Begin+Window−2) {HPhob = OMH (protein(Pos) );}
   HydSum = HydSum+HPhob;
   for (AngPos = 1,NumAngle)
   {
    Angle = RadAngle(AngPos) * (Pos);
    SinSum(AngPos) = SinSum(AngPos)+Sin(Angle) * HPhob;
    CosSum(AngPos) = CosSum(AngPos)+Cos(Angle) * HPhob;
    CorSSum(AngPos) = CorSSum(AngPos)+Sin(Angle);
    CorCSum(AngPos) = CorCSum(AngPos)+Cos(Angle);
   }
   }
- Calculate the moment on each window by adding the new position to the sine and cosine sums, and subtracting the old.
   for (Pos = Begin+Window−1, Fin+Window−1 )
   {
    MaxValue(Pos−Window+1) = 0;
    MaxAngle(Pos−Window+1) = 0;
    HPhob = OMH( protein(Pos) );
    HydSum = HydSum+HPhob;
    HAve = HydSum/Window;
- Add the new value at the right end of the window.
   for (AngPos = 1, NumAngle)
   {
    Angle = RadAngle(AngPos) * (Pos);
    SinSum(AngPos) = SinSum(AngPos)+Sin(Angle) * HPhob;
    CosSum(AngPos) = CosSum(AngPos)+Cos(Angle) * HPhob;
    CorSSum(AngPos) = CorSSum(AngPos)+Sin(Angle);
    CorCSum(AngPos) = CorCSum(AngPos)+Cos(Angle);
- Calculate the moment, and save the max.
   Moment = Sqrt( (SinSum(AngPos)−CorSSum(AngPos)*HAve)^2^+
    (CosSum(AngPos)−CorCSum(AngPos)*HAve)^2^ );
   Moment = Moment/Window;
   If (Moment>MaxValue(Pos−Window+1))
   {
    MaxValue(Pos−Window+1) = Moment;
    MaxAngle(Pos−Window+1) = StartAngle+(AngPos−1)*IncAngle;
   }
  }
- Substract the oldest value from the left end of the window.
   HPhob = OMH(protein(Pos−Window+1) );
   HydSum = HydSum−Hphob;
   for ( AngPos = 1, NumAngle )
   {
    Angle = RadAngle(AngPos) * (Pos−Window+1);
    SinSum(AngPos) = SinSum(AngPos)−Sin(Angle) * HPhob;
    CosSum(AngPos) = CosSum(AngPos)−Cos(Angle) * HPhob;
    CorSSum(AngPos) = CorSSum(AngPos)−Sin(Angle);
    CorCSum(AngPos) = CorCSum(AngPos)−Cos(Angle);
   }
  }

Thus, the propensity of a peptide to be unstructured is predicted by MH and MC, the charge is accounted by the IP and the amphipathicity by the μH.

The proposed algorithms were first analyzed from its original implementation in FORTRAN77 and tested on a cluster of four computers with each a Pentium IV at 2.4 GHz running on Linux.

To evaluate the number of unstructured peptides predicted by the AGADIR score and the charge and mean hydrophobicity criteria (C(MH), see equation 2), 1.6×10^5^ peptide sequences (20^4^) of 9 amino acids in length were evaluated corresponding to sequences derived from the following pattern: RAAAYXXXX, where letter X stands for any of the 20 amino acid, Y, R and A stand for Tyrosine, Arginine and Alanine, respectively. The same sequences were used to evaluate the time efficiencies obtained in our FPGA board.

The following steps were performed for the physical implementation of the Handel-C code into the FPGA:

1. The Handel-C code is translated to VDHL code using the DK5 Handel-C Compiler.
2. The generated VHDL code is embedded as a component into a VHDL project, which contains an I/O interface.
3. Then, the Synthesis, Map and Place and Route processes from the Xilinx Tools (Xilinx ISE 9.2i) are executed. Finally, the configuration file for the target FPGA device is generated and downloaded to the FPGA board for testing.

To determine the execution time per peptide sequence, we recorded the total time reported in the hardware and software implementations and divided by the number of peptide sequences analyzed; this is reported as the average time for each implementation. Not every sequence in the 1.6×10^5^ peptide sequences analyzed had features of known SCAPs, and consequently not every algorithm computed every peptide sequence. Thus, four parallel counters were implemented to account for the execution time of each hardware module. The values stored in these counters represent the total number of cycles required for each hardware process. To obtain the total execution time in hardware of each process, the value of each counter is multiplied by the reciprocal of the clock frequency, in this case 50 MHz because this frequency is lower than the maximum clock frequency possible for the design.

## Results

### Software Adaptation

In this work, we predict SCAPs by computing the charge (Isoelectric Point, pI), amphipathicity (Helical Hydrophobic Moment, μH) and the propensity of a peptide sequence to be natively unfolded by combining two physicochemical properties. This last property was originally estimated using the AGADIR score [9]; however, we noted that for short peptides the AGADIR code does not discriminate efficiently. Specifically, we observed than in 1.6×10^5^ sequences of 9 amino acids in length generated with an infinite period random algorithm built on a unit square and evaluated with AGADIR, only 620 of these sequences (0.0388%) did not have an AGADIR score within the range of known SCAPs (AGADIR<10).

**Figure 1:**
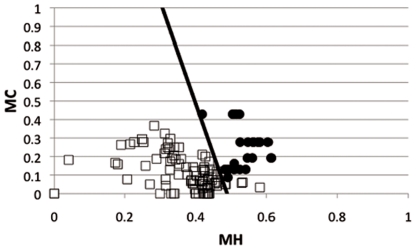
Predicting unstructured antibacterial peptides. Two physicochemical properties (Mean Charge, MC, Mean Hydrophobicity, MH) known to differentiate structured from non-structured proteins were applied to antibacterial peptides. The figure shows the paired values of a set of structured antibacterial peptides (empty squares) and unstructured antibacterial peptides (filled circles). See supplementary Table 1 for further information about the peptide sequences used for this study.

Thus, here we report the use of a simpler algorithm that has been previously described to predict natively unfolded proteins [16]; for that we calculated the Mean Charge (MC) and the Mean Hydrophobicity (MH). Figure 1 and Table S1 show the values obtained for these physicochemical properties in antibacterial peptides reported to be structured and non-structured. Note that these two sets of peptides are separated roughly with the line (linear equation 2) plotted in the figure.

### FPGA architecture

**Figure 2:**
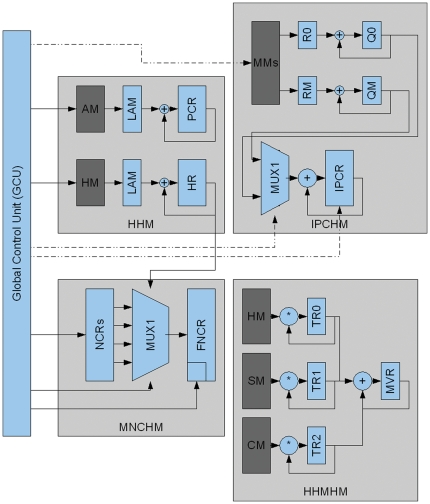
FPGA functional architecture. The 4 hardware modules required to predict SCAPs (Mean Charge, MNCHM, Mean Hydrophobicity, HHM, Isoelectric Point, IPCHM, Hydrophobic Moment, HHMHM) were integrated into an FPGA board as depicted: If MNCHM and HHM were consistent with values of known SCAP (see Eqn. 2 in Methods), then IPCHM was calculated; if IPCHM was within the values of known SCAP the HHMHM was finally calculated.

We divided the prediction of each peptide in 4 basic blocks, where each block performs each one of the 4 physicochemical properties calculations (see Design and Implementation section). A functional architecture of this design is described in Figure 2. This architecture is based on the computation demand of each module observed in the software and hardware version (see Table 1, column labelled “Execution Time (Software)”), minimizing the use of the most demanding one (μH>pI>MC>MH).

**Table 1:** Performance statistics.

Algorithm	Execution Time (Hardware)	Execution Time (Software)
MC	0.107 ms	14.39 ms
MH	0.157 ms	14.39 ms
pI	0.157 ms	30.76 ms
μH	14.5 ms	249.2 ms
Average	5.15 ms	23.61 ms

The average and individual execution time per peptide sequence and algorithm implemented in the FPGA card and the original version in software (Fortran77) running on a Linux box is reported (see Methods). The reported time for the software version was derived using a cluster of 4 Pentium IV 2.4 GHz. The time reported for the software version corresponds to the actual user time as measured by the time command in the Linux box. The time reported for the Hardware version corresponds to the one reported by the Xilinx tools (see Methods).

The hardware architecture modules include:

- Hydrophobicity Hardware Module (HHM), implements the MH algorithm in four storage modules (Amino acids Memory (AM), hydrophobicity Memory (HM), Peptide Charge Register (PCR) and Hydrophobicity Register (HR)) and one Length Adjusting Module (LAM).
- Mean Net Charge Hardware Module (MNCHM), implements the MC algorithm using a set of registers required to accelerate this calculation (Net Charge Register, NCR); the final values were registered in the FNCR memory of this module.
- Isoelectric Point Charge Hardware Module (IPCHM), implements the pI algorithm; for this, several multilevel memory access in parallel are used to accelerate the computation (temporal registers R0, R1, … RM) that is stored ultimately at the Isoelectric Point Charge Register (IPCR).
- Helical Hydrophobic Moment Hardware Module (HHMHM) implements the μH algorithm. This module actually computes the MC and MH values that are used by the MNCHM; it stores also the Sine (SM) and Cosine (CM) values that accelerate the μH calculation; however, this acceleration is counterbalanced by the large number of cycles required to execute this module. The values in the HM, SM and CM are stored in three temporal registers (TR0, TR1 and TR2). The final result is stored in the Mean Value Register (MVR).
- Global Control Unit (GCU) defines the execution order of each of the hardware modules; this is currently achieved by activating one module at a time changing the control signal of the Multiplexer1 (MUX1) in each hardware module.

Every peptide sequence was represented by a fixed-point number and generated by the FPGA board, thus eliminating the overhead due to the CPU-FPGA data communication. We validated our peptide representation in the FPGA comparing the results obtained using the FPGA with the software version (see Design and Implementation section) and observed 100% match in all the tested sequences: from the 1.6×10^5^ peptide sequences analyzed, the same 4,984 peptide sequences were predicted as SCAPs by our FPGA board and the software version.

In total, our FPGA implementation uses up to 99% of logic and RAM memory and 5% of input/output resources of the FPGA itself simulated at a maximum frequency of 55.16 MHz/cycles (see Table 2). Since it is not practically possible to set the clock speed at that frequency, we will use the immediate possible lower value, 50 MHz, for our analysis.

**Table 2:** Hardware utilization statistics.

	MC	MH	pI	μH	Complete System
Flip Flops	444 (2%)	704 (3%)	1,037 (4%)	1,518 (6%)	3,703 (13%)
Look-up Tables	1,224 (4%)	1,935 (7%)	2,857 (10%)	4,183 (15%)	10,202 (37%)
Slices	711 (5%)	1,125 (8%)	1,685 (12%)	2,428 (18%)	5,923 (43%)
Gates	105,640	167,263	246,492	360,935	880,329
Block RAMs	-	-	2 (1%)	11 (8%)	13 (10%)
MULT18×18s	-	-	1 (1%)	10 (7%)	11 (8%)
Max Clock Frequency	75.34 Mhz	75.30 Mhz	73.04 Mhz	67.51 MHz	55.16 Mhz

The table reports the resources used by the 4 codes (MC, MH, pI, μH) used to predict SCAPs when implemented on a Xilinx Virtex II PRO family FPGA board.

All the physicochemical values use floating-point operations, but for our FPGA implementation these were estimated as fixed-point integer numbers. The floating-point arithmetic circuits require more space than integer representation in the FPGA. Hence, integer representation is preferred to maximize the area used in the FPGA device and allow for parallelism. In this way, each one of the 20 amino acids was represented by an integer from 0 to 19; each integer is coded in 5 bits using a binary representation: *e.g.*, the decimal number *19* is represented by 5 bits in a binary code, *10011*. Thus, for the peptides of 9 amino acids in length used in this study, we used up to 45-bits for each peptide. To generate all the peptide sequences tested in the FPGA card, we used a simple counter.

Each module was optimized in order to return one result per clock cycle. With a 50 MHz clock frequency, the execution times achieved are reported in Table 1. The optimized version of this program on the FPGA card takes on average 5.15 µs to evaluate each peptide sequence; the same program on a Linux box at 2.4 GHz takes on average 23.6 µs, thus the FPGA implementation is 4.5 times faster on average. Note that the sum of the execution time of each algorithm does not add up to the reported average execution time, because in our implementation not every code is executed per sequence (see Figure 2 and Methods). As noted in Table 2, the best improvement in performance was achieved in the pI calculation module, which execution time was 195 times faster in the FPGA device and included a parallel routine.

## Discussion

There are different software solutions to compute physicochemical properties of peptides. Despite the high-performance computing nature of these calculations, there have been no developments to solve these in an efficient way. The FPGA's features are adequate to address the computation of physicochemical properties of peptides, because these allow testing diverse embedded codes at low cost and relatively short developing times. Additionally, having a custom FPGA implementation of these algorithms is important based on:

- Our interest to exhaustively explore the sequence space of peptides to identify potential SCAPs,
- While FPGA performance may compete with clusters of computers, the cost of running and maintaining FPGA platforms is importantly lower than those of computer clusters and
- There are many physicochemical properties used in the prediction of antibacterial peptides [17] and other bioinformatics applications (*e.g.*, predictions of proteins natively unfolded) that could benefit from the description of this work.

Here we report an optimization and implementation into an FPGA device of four instantiations of algorithms useful to compute physicochemical properties of peptides. FPGA boards are a convenient platform to build custom computing processors and achieve high performance at a fraction of the cost of other high-performance computing solutions. FPGAs are attracting the attention of scientists in the bioinformatics area, and different approaches to design and program them are available (*e.g.*, Mentor [18], Mitrionics [19]). The key acceleration component of FPGAs is the parallelization of the algorithm. Problems like the one exposed in this work, can be massively parallelized, gaining orders of magnitude in performance (*e.g.*, the pI algorithm, see Table 1). The limitation is the actual physical space in the FPGA device.

Note that 3 (net charge, mean hydrophobicity and isoelectric point) out of 4 physicochemical properties computed are insensitive to the amino acid order in the peptide sequence, but the helical hydrophobic moment is not. If all the properties would have this feature, only a fraction of all possible peptide sequences would be analyzed and there would be no need for an FPGA implementation. However, as noted in Table 1, the helical hydrophobic moment takes up 80% of the computing time, so the FPGA implementation is required.

In our study, we show that our code has the same precision than the software version of it. Yet, the size of the four codes used up to 99% of the logic and RAM memory, preventing further replication in the tested FPGA card. However, the reported number of gates per algorithm in Table 2 (0.8 million gates) may allow estimating the gain of speed by replicating these codes in a denser FPGA card. For instance, in a Virtex-6 FPGA device with 8 millions gates, a potential performance improvement would be obtained by replicating each module 10 times. Thus, in such FPGA board the code will execute 45 times faster only due to parallelism, compared with software running at the PC computer. Furthermore, using a Virtex-6 device, our FPGA implementation could gain in clock speed since Virtex-6 devices have logic elements that support up to 500 MHz, compared to 100 MHz of the testing platform. Therefore, with a conservative clock speed increase of 200 MHz for the implementation, an overall possible acceleration with Virtex-6 could be in the order of 100–120 times compared to the PC software version. Extending the architecture for larger FPGA devices or hives of FPGA boards operating in parallel, may require some extensions to the architecture to manage several execution threads, but it could further accelerate processing time.

Besides the overall acceleration of our FPGA implementation, individually each of the 4 physicochemical properties calculations were accelerated from 17 to 195 times (see Table 1) being the implementation of the pI code the one with the largest acceleration due to its parallelized implementation. It is important to note that a gain in time performance is expected when the embedded code is parallelized, yet it is not possible to anticipate the magnitude of the acceleration since it depends on the nature of each algorithm.

Another important aspect in the development of FPGA codes is the time involved in coding low-level routines in FPGA boards. To accelerate this, we used the Handel-C language, which is based on the ANSI-C standard but with explicit parallelization constructs. Since coding in Handel-C has to be done keeping in mind the physical restrictions of the FPGA device, the designer has to consider the number of bits of every variable, types of arithmetic representation and operations. During the compilation and hardware synthesis, the code in Handel-C is transformed into a VHDL language representation. Thus, a second inspection of the code is always needed according to the FPGA platform model used.

Finally, it is important to note that there are many other physicochemical properties that may be relevant to embed on dedicated hardware. As more of these routines became available, these may be treated as modules on FPGA boards (*e.g.*, as the hardware blocks described here) for different simulation purposes. For instance, FPGA boards capable to compute physicochemical properties may be used to analyze natively unfolded peptide sequences and SCAPs in an exhaustive fashion, among others. Our results may pave the way towards that goal.

### Conclusions

In summary, we report an implementation of an FPGA card with 4 embedded codes useful in the exhaustive prediction of physicochemical properties of peptides, particularly in the prediction of selective cationic antibacterial peptides.

### Availability and Future Directions

We have made available the code at sourceforge within the project named APAP-FPGA in the following address: http://apap-fpga.sourceforge.net/

The actual code is made available under the GNU GPL v3 license at:

http://apap-fpga.svn.sourceforge.net/viewvc/apap-fpga/

Any changes and improvements on this code will be reflected on this web site.

## Supporting Information

Table S1 **Structured and unstructured antibacterial peptides.** Antibacterial peptide sequences used to test the method to discriminate structured from non-structured peptides. **ID** refers to the number identification reported in the Antimicrobial Peptide Database (Wang, Z. and Wang, G. (2004) APD: the Antimicrobial Peptide Database. Nucleic Acids Research 32, D590–D592); NS indicated that the peptide sequence is Not Specified in the APD and were obtained elsewhere (del Rio G, Castro-Obregon S, Rao R, Ellerby HM, Bredesen DE. 2001. APAP, a sequence-pattern recognition approach identifies substance P as a potential apoptotic peptide. FEBS Lett. 494:213–219); **Sequence** reports the corresponding peptide sequence using the single-letter amino acid code. The antibacterial peptides reported to be non-structured in water solution are in indicated with a gray background and the structured ones are in white cells in the table.
(DOC)
