# Supplementary material for: An FPGA Implementation to Detect Selective Cationic Antibacterial Peptides
Source: PLoS One. 2011 Jun 28;6(6):e21399. doi: 10.1371/journal.pone.0021399 (PMC3125173; doi:10.1371/journal.pone.0021399)
Supplement: Table S1 — Structured and unstructured antibacterial peptides. Antibacterial peptide sequences used to test the method to discriminate structured from non-structured peptides. ID refers to the number identification reported in the Antimicrobial Peptide Database (Wang, Z. and Wang, G. (2004) APD: the Antimicrobial Peptide Database. Nucleic Acids Research 32, D590–D592); NS indicated that the peptide sequence is Not Specified in the APD and were obtained elsewhere (del Rio G, Castro-Obregon S, Rao R, Ellerby HM, Bredesen DE. 2001. APAP, a sequence-pattern recognition approach identifies substance P as a potential apoptotic peptide. FEBS Lett. 494:213–219); Sequence reports the corresponding peptide sequence using the single-letter amino acid code. The antibacterial peptides reported to be non-structured in water solution are in indicated with a gray background and the structured ones are in white cells in the table. (DOC) [file pone.0021399.s001.doc]

### Table S1 – Structured and unstructured antibacterial peptides

| **ID** | **Sequence** |
| --- | --- |
| 00001 | GLWSKIKEVGKEAAKAAAKAAGKAALGAVSEAV |
| 00047 | GPLSCGRNGGVCIPIRCPVPMRQIGTCFGRPVKCCRSW |
| 00055 | IIGPVLGMVGSALGGLLKKI |
| 00072 | VIPFVASVAAEMQHVYCAASRKC |
| 00088 | GILDTLKQFAKGVGKDLVKGAAQGVLSTVSCKLAKTC |
| 00094 | FLPLIGRVLSGIL |
| 00102 | GSKKPVPIIYCNRRTGKCQRM |
| 00103 | DKLIGSCVWGAVNYTSDCNGECLLRGYKGGHCGSFANVNCWCET |
| 00134 | SWLSKTAKKLENSAKKRISEGIAIAIQGGPR |
| 00140 | SQLGDLGSGAGQGGGGGGSIRAAGGAFGKLEAAREEEFFYKKQKEQLERLKNDQIHQAEFHHQQIKEHEEAIQRHKDFLNNLHK |
| 00142 | GLKKLLGKLLKKLGKLLLK |
| 00143 | KKLLKWLKKLL |
| 00145 | VNYGNGVSCSKTKCSVNWGQAFQERYTAGINSFVSGVASGAGSIGRRP |
| 00147 | AKIPIKAIKTVGKAVGKGLRAINIASTANDVFNFLKPKKRKA |
| 00149 | MPCSCKKYCDPWEVIDGSCGLFNSKYICCREK |
| 00151 | RCVCTRGFCRCVCRRGVC |
| 00153 | RSVCRQIKICRRRGGCYYKCTNRPY |
| 00154 | YSRCQLQGFNCVVRSYGLPTIPCCRGLTCRSYFPGSTYGRCQRY |
| 00155 | RGLRRLGRKIAHGVKKYGPTVLRIIRIAG |
| 00156 | RCVCRRGVCRCVCRRGVC |
| 00166 | GWGSFFKKAAHVGKHVGKAALTHYL |
| 00170 | VDKGSYLPRPTPPRPIYNRN |
| 00172 | GKPRPYSPRPTSHPRPIRV |
| 00193 | DTHFPICIFCCGCCHRSKCGMCCKT |
| 00194 | VGECVRGRCPSGMCCSQFGYCGKGPKYCGR |
| 00197 | KNLRRIIRKGIHIIKKYG |
| 00198 | KNLRRITRKIIHIIKKYG |
| 00199 | KYYGNGVHCTKSGCSVNWGEAFSAGVHRLANGGNGFW |
| 00205 | ITSISLCTPGCKTGALMGCNMKTATCHCSIHVSK |
| 00210 | GMASKAGAIAGKIAKVALKAL |
| 00216 | ATCDLLSGTGINHSACAAHCLLRGNRGGYCNGKGVCVCRN |
| 00227 | ATCDLLSGTGINHSACAAHCLLRGNRGGYCNGKAVCVCRN |
| 00230 | GWLKKIGKKIERVGQHTRDATIQGLGIAQQAANVAATAR |
| 00235 | NPVSCVRNKGICVPIRCPGSMKQIGTCVGRAVKCCRKK |
| 00281 | GLLRKGGEKIGEKLKKIGQKIKNFFQKLVPQPEQ |
| 00285 | GLLCYCRKGHCKRGERVRGTCGIRFLYCCPRR |
| 00369 | RIIDLLWRVRRPQKPKFVTVWVR |
| 00394 | QVYKGGYTRPIPRPPPFVRPLPGGPIGPYNGCPVSCRGISFSQARSCCSRLGRCC |
| 00402 | KTCENLANTYRGPCFTTGSCDDHCKNKEHLRSGRCRDDFRCWCTRNC |
| 00403 | ACNFQSCWATCQAQHSIYFRRAFCDRSQCKCVFVRG |
| 00414 | SIGSALKKALPVAKKIGKIALPIAKAALP |
| 00418 | GLRKRLRKFRNKIKEKLKKIGQKIQGFVPKLAPRTDY |
| 00420 | HSSGYTRPLRKPSRPIFIRPIGCDVCYGIPSSTARLCCFRYGDCCHL |
| 00428 | SAFTVWSGPGCNNRAERYSKCGCSAIHQKGGYDFSYTGQTAALYNQAGCSGVAHTRFGSSARACNPFGW |
| 00429 | GYFCESCRKIIQKLEDMVGPQPNEDTVTQAASQVCDKLKILRGLCKKIMRSFLRRISWDILTGKKPQAICV |
| 00434 | GLMSVLGHAVGNVLGGLFKS |
| 00437 | EFTNVSCTTSKECWSVCQRLHNTSRGKCMNKKCRCYS |
| 00438 | GFGCPNNYQCHRHCKSIPGRCGGYCGGWHRLPCTCYRCG |
| 00451 | DHYNCVSSGGQCLYSACPIFTKIQGTCYRGKAKCCK |
| 00480 | VGIGTPIFSYGGGAGHVPEYF |
| 00484 | RGFRKHFNKLVKKVKHTISETAHVAKDTAVIAGSGAAVVAAT |
| 00485 | GFGALFKFLAKKVAKTVAKQAAKQGAKYVVNKQME |
| 00499 | VGALAVVVWLWLWLW |
| 00505 | DSHAKRHHGYKRKFHEKHHSHRGY |
| 00507 | GLLSKVLGVGKKVLCGVSGLC |
| 00513 | FLGGLIKIVPAMICAVTKKC |
| 00524 | GIGDPVTCLKSGAICHPVFCPRRYKQIGTCGLPG |
| 00546 | FLSLIPHAINAVSAIAKHN |
| 00548 | RFGRFLRKIRRFRPKVTITIQGSARFG |
| 00554 | GKIPVKAIKKAGAAIGKGLRAINIASTAHDVYSFFKPKHKKK |
| 00555 | KGRGKQGGKVRAKAKTRSS |
| 00558 | GFGCPGNQLKCNNHCKSISCRAGYCDAATLWLRCTCTDCNGKK |
| 00562 | GKVWDWIKSAAKKIWSSEPVSQLKGQVLNAAKNYVAEKIGATPT |
| 00592 | GILSSFKGVAKGVAKDLAGKLLETLKCKITGC |
| 00613 | RVKRFWPLVPVAINTVAAGINLYKAIRRK |
| 00630 | GEILCNLCTGLINTLENLLTTKGADKVKDYISSLCNKASGFIATLCTKVLDFGIDKLIQLIEDKVDANAI |
| 00633 | KYYGNGVHCGKHSCTVDWGTAIGNIGNNAAANWATGGNAGWNK |
| 00637 | ARSYGNGVYCNNKKCWVNRGEATQSIIGGMISGWASGLAGM |
| 00644 | GFFALIPKIISSPLFKTLLSAVGSALSSSGGQE |
| 00707 | RLFDKIRQVIRKF |
| 00727 | RWCVYAYVRVRGVLVRYRRCW |
| 00731 | SFGLCRLRRGFCARGRCRFPSIPIGRCSRFVQCCRRVW |
| 00757 | FLSLIPHAINAVSTLVHHF |
| 00758 | FLSLIPHAINAVSALANHG |
| 00764 | GLRSKIWLWVLLMIWQESNKFKKM |
| 00780 | GRRRRSVQWCAVSQPEATKCFQWQRNMRKVRGPPVSCIKRDSPIQCIQA |
| 00855 | RCVCTRGFCRCICLLGIC |
| 00899 | FLPIVTNLLSGLL |
| 00900 | FLSHIAGFLSNLF |
| 00913 | QWGRRCCGWGPGRRYCVRWC |
| 00928 | NKGCATCSIGAACLVDGPIPDFEIAGATGLFGLWG |
| 00929 | MAKEFGIPAAVAGTVINVVEAGGWVTTIVSILTAVGSGGLSLLAAAGRESIKAYLKKEIKKKG |
| 01005 | YVSCLFRGARCRVYSGRSCCFGYYCRRDFPGSIFGTCSRRNF |
| 01006 | YITCLFRGARCRVYSGRSCCFGYYCRRDFPGSIFGTCSRRNF |
| 01007 | KWKLFKKIGAVLKVL |
| 01151 | GTWDDIGQGIGRVAYWVGKALGNLSDVNQASRINRKKKH |
| 01153 | YLAFRCGRYSPCLDDGPNVNLYSCCSFYNCHKCLARLENCPKGLHYNAYLKVCDW |
| 01154 | AIKLVQSPNGNFAASFVLDGTKWIFKSKYYDSSKGYWVGIYEVWDRK |
| 01158 | ALYKKFKKKLLKSLKRL |
| 01168 | LVAYGIAQGTAEKVVSLINAGLTVGSIISILGGVTVGLSGVFTAVKAAIAKQG |
| 01177 | FNRGGYNFGKSVRHVVDAIGSVAGILKSIR |
| 01194 | CSTNTFSLSDYWGNNGAWCTLTHECMAWCK |
| 01206 | CTFTLPGGGGVCTLTSECIC |
| 01224 | VGALAVVVWLFLWLW |
| 01225 | VGALAVVVWLYLWLW |
| 01227 | VGIGGGGGGGGGGSCGGQGGGCGGCSNGCSGGNGGSGGSGSHI |
| 01238 | NPLIPAIYIGATVGPSVWAYLVALVGAAAVTAANIRRASSDNHSCAGNRGWCRSKCFRHEYVDTYYSAVCGR |
| 01239 | KFFRKLKKSVKKRAKEFFKKPRVIGVSIPF |
| 01249 | GILDAIKAIAKAAG |
| 01267 | RRTCHCRSRCLRRESNSGSCNINGRIFSLCCR |
| 01381 | DDTPSSRCGSGGWGPCLPIVDLLCIVHVTVGCSGGFGCCRIG |
| 01471 | RPKPQQFFGLM |
| 00139 | KWKLFKKIEKVGQNIRDGIIKAGPAVAVVGQATQIAK |
| 00144 | GIGKFLHSAKKFGKAFVGEIMNS |
| 00146 | GIGAVLKVLTTGLPALISWIKRKRQQ |
| NS | KIAKKIAKIAKKIA |
| NS | KIAKKIAKIAKKIAKIAKKIA |
| NS | KIAKLAKKIAKLAK |
| NS | KIAKLAKKIAKLAKKIAKLAK |
| NS | KALKALKKALKALKKALKALK |
| NS | KLGKKLGKLGKKLGKLGKKLG |
| NS | KWKLFKKIEKVGQGIGAVLKVLTTGL |
| NS | KWKLFKKIGIGAVLKVLTTGLPALIS |
| NS | KLALKLALKAWKAALKLA |
| NS | KLALKAALKAWKAAAKLA |
| NS | KLALKAAAKAWKAAAKAA |
| NS | KAIAKSILKWIKSIAKAI |
| NS | KALAALLKKWAKLLAALK |
| NS | KITLKLAIKAWKLALKAA |
| NS | KALAKALAKLWKALAKAA |
| NS | GIGKFLHSAKKFGKAWVGEIMNS |
| NS | GLGKFLHSAKRFGKAFVGEAMNS |
| NS | GIGKFIHSAKKFGKLFVGEIMNS |
| NS | GIGKFIHAAKKFGKLFIGEIMNS |
| NS | GIGKFIHSAKRFGRAWVGEIMNS |
| NS | GIGKFIHSVKKWGKTFIGEIMNS |
| NS | GIAKFGKAAAHFGKKWVGELMNS |
| NS | GIGKFLHTLKTFGKKWVGEIMNS |
| NS | GIGHFLHKVKSFGKSWIGEIMNS |
